# Contribution of TMS and rTMS in the Understanding of the Pathophysiology and in the Treatment of Dystonia

**DOI:** 10.3389/fncir.2016.00090

**Published:** 2016-11-10

**Authors:** Pierre Lozeron, Aurélia Poujois, Alexandra Richard, Sana Masmoudi, Elodie Meppiel, France Woimant, Nathalie Kubis

**Affiliations:** ^1^Service de Physiologie Clinique-Explorations Fonctionnelles, AP-HP, Hôpital LariboisièreParis, France; ^2^INSERM UMR965Paris, France; ^3^Sorbonne Paris Cité – Université Paris DiderotParis, France; ^4^Service de Neurologie, AP-HP, Hôpital LariboisièreParis, France; ^5^Centre de Référence National de la Maladie de Wilson, Hôpital LariboisièreParis, France

**Keywords:** dystonia, transcranial magnetic stimulation, basal ganglia, plasticity, surround inhibition, integration

## Abstract

Dystonias represent a heterogeneous group of movement disorders responsible for sustained muscle contraction, abnormal postures, and muscle twists. It can affect focal or segmental body parts or be generalized. Primary dystonia is the most common form of dystonia but it can also be secondary to metabolic or structural dysfunction, the consequence of a drug’s side-effect or of genetic origin. The pathophysiology is still not elucidated. Based on lesion studies, dystonia has been regarded as a pure motor dysfunction of the basal ganglia loop. However, basal ganglia lesions do not consistently produce dystonia and lesions outside basal ganglia can lead to dystonia; mild sensory abnormalities have been reported in the dystonic limb and imaging studies have shown involvement of multiple other brain regions including the cerebellum and the cerebral motor, premotor and sensorimotor cortices. Transcranial magnetic stimulation (TMS) is a non-invasive technique of brain stimulation with a magnetic field applied over the cortex allowing investigation of cortical excitability. Hyperexcitability of contralateral motor cortex has been suggested to be the trigger of focal dystonia. High or low frequency repetitive TMS (rTMS) can induce excitatory or inhibitory lasting effects beyond the time of stimulation and protocols have been developed having either a positive or a negative effect on cortical excitability and associated with prevention of cell death, γ-aminobutyric acid (GABA) interneurons mediated inhibition and brain-derived neurotrophic factor modulation. rTMS studies as a therapeutic strategy of dystonia have been conducted to modulate the cerebral areas involved in the disease. Especially, when applied on the contralateral (pre)-motor cortex or supplementary motor area of brains of small cohorts of dystonic patients, rTMS has shown a beneficial transient clinical effect in association with restrained motor cortex excitability. TMS is currently a valuable tool to improve our understanding of the pathophysiology of dystonia but large controlled studies using sham stimulation are still necessary to delineate the place of rTMS in the therapeutic strategy of dystonia. In this review, we will focus successively on the use of TMS as a tool to better understand pathophysiology, and the use of rTMS as a therapeutic strategy.

## Introduction

Dystonia is an involuntary movement defined by sustained muscle contraction, abnormal postures and muscle twists (Fahn, 1988). It represents a heterogeneous group of syndromes that has been initially considered as secondarily to basal ganglia dysfunction, based on the pathological and the early brain imaging studies. However, the results of new imaging and functional investigations suggest that the pathophysiology of dystonia is more complex and involves other brain structures such as the cerebellum and various cortical areas.

Because there is no appropriate animal model (Raike et al., 2005), pathophysiological mechanisms remained speculative before the era of TMS. By modulation of the cortical activity after the application of a magnetic current over the cortex, rTMS allowed to further our understanding of the mechanisms taking place in the various dystonic syndromes.

Treatment of dystonia is symptomatic and partially effective. It relies mainly on botulinum toxin injections that must be repeated in the tonic muscles. Several medications such as levodopa, anticholinergic and antiepileptic drugs have been tested without consistent efficacy. For the most severe cases, surgical pallidotomy or thalamotomy has been proposed but is now abandoned, as the resulting lesions are irreversible. A functional lesion by deep brain stimulation of the internal portion of the globus pallidus is a potential alternative treatment but remains an invasive procedure, so that other treatments are looked at (Albanese et al., 2015). In the past years, TMS allowed to improve the understanding of the pathophysiology of dystonia by the evaluation of various cortical areas’ excitability. Furthermore, the potential benefit of cortical excitability modulation by rTMS in the treatment of dystonia has been evaluated with promising results based on the few studies reported to date.

In this review, after giving the definition and classification of dystonia, we describe its presumed pathophysiology in order to understand how TMS has been used to go deeper into the comprehension of the pathophysiology of the disease. Then, we report how rTMS protocols have been developed as a therapeutic tool in dystonia, and compare rTMS to other non-invasive brain stimulation techniques.

## Dystonia: Definition and Anatomical Structures Impairment

Dystonia has been classified not only by its clinical characteristics including age at onset, distribution of symptoms (focal, segmental, multifocal, hemidystonia, or generalized), temporal pattern (persistent, action specific, diurnal fluctuations, or permanent) and associated features (isolated dystonia or combined dystonia associated with systemic or other neurological manifestations) but also by etiology including inherited (autosomal dominant most often due to the deletion of a GAG trinucleotide in the *DYT1* gene linked to the chromosome 9q32-34 (Kramer et al., 1990), autosomal recessive, X-linked recessive or mitochondrial), acquired (perinatal brain injury, infection, drug, toxic, vascular, neoplastic, brain injury, or psychogenic) and idiopathic (sporadic, familial, other) (Albanese et al., 2013).

Compared to previous concepts classifying dystonia as primary and secondary, this new classification offers the advantage to describe more precisely various aetiologies. Nevertheless, the classification between primary and non-primary dystonia is still used in many studies to describe patients without or with brain lesion.

Dystonia is associated with co-contraction of antagonist muscles and contraction of adjacent unnecessary muscles to the movement that refer to overflow. Thus, overflow, defined as an unintentional muscle contraction, which accompanies, but is anatomically distinct from the primary voluntary or involuntary movement, is a characteristic of the disease (Ceballos-Baumann et al., 1995a; Geyer and Bressman, 2006). It affects almost 50% of the patients with writer’s cramp (Jedynak et al., 2001). In idiopathic torsion dystonia, persistent electromyography (EMG) activity in agonist and antagonist muscles has been recorded (Yanagisawa and Goto, 1971) even during stretching of contralateral muscles. The pathophysiology of dystonia is not clearly understood but a unique mechanism is doubtful given the heterogeneity of dystonia syndromes. Neuronal loss is rarely reported in the few autopsy cases of focal, segmental or generalized dystonia (Gibb et al., 1988; Zweig et al., 1988). Patchy neuronal loss and gliosis has been rarely found in the caudate nucleus or in the putamen (especially in their dorsal parts) in idiopathic generalized dystonia (Gibb et al., 1992), or in the cerebellum in cervical dystonia (Kostić et al., 1996; Prudente et al., 2013).

**FIGURE 1:**
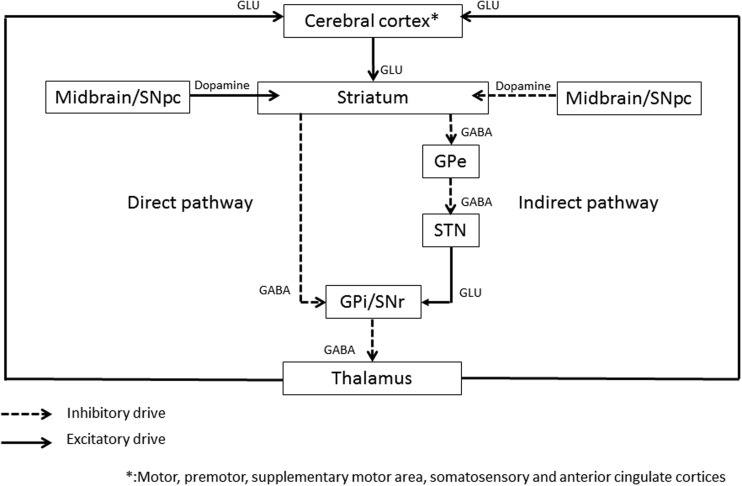
**Schematic representation of the basal ganglia circuitry.** GPi, Globus Pallidus internus; GPe, Globus Pallidus externus; STN, subthalamic nucleus; SNr, substantia nigra pars reticulata; GLU, glutamate; GABA, gamma-aminobutyric acid. The following areas are target areas to improve dystonia with rTMS: primary cortex, premotor and supplementary motor cortex, somatosensory cortex (SSC), anterior cingulate cortex (ACC), and cerebellar cortex.

The central role of basal ganglia loop (**Figure 1**) in dystonia has been supported by the beneficial results of thalamotomy (Andrew et al., 1983; Vitek et al., 1998), pallidotomy (Ondo et al., 1998), or basal ganglia deep brain stimulation (Vidailhet et al., 2005; Kupsch et al., 2006; Volkmann et al., 2012) on dystonic symptoms. In the literature, the dysfunctionning of basal ganglia has been largely assessed by means of a wide range of functional imaging tools. GMV, evaluated by voxel-based morphometry (VBM) by averaging high-resolution MRI whole brain images (Draganski et al., 2004), has shown either an increase or a decrease of the putamen (Etgen et al., 2006; Obermann et al., 2007; Granert et al., 2011), the globus pallidus (Draganski et al., 2003; Granert et al., 2011), or the caudate (Obermann et al., 2007) in primary focal dystonia. fMRI, which detects the blood oxygen level-dependant changes during a specific task, thus reflecting the blood flow changes associated with brain activity, has shown a relative increased activity in the basal ganglia. At last, ^18^F-fluorodeoxyglucose PET, which evaluates the areas of brain activity based on glucose metabolism, has shown a bilateral increased activity in the lentiform nucleus at rest in childhood onset dystonia (Eidelberg et al., 1998).

However, as onset of dystonia can be postponed after a basal ganglia lesion (Pettigrew and Jankovic, 1985) and as clinical response of the dystonic symptoms can be delayed after pallidotomy (Ondo et al., 1998) or deep brain stimulation (Alterman and Snyder, 2007), it suggests that lesions of the basal ganglia alone cannot fully explain the disorder. Indeed, radiological lesions of basal ganglia on standard brain imaging do not consistently induce dystonia (Bhatia and Marsden, 1994), and lesions outside the basal ganglia have been reported in cervical dystonia or hemidystonia (Krauss et al., 1991; Krauss et al., 1997; LeDoux and Brady, 2003) and in experimental rats’ studies, the association of another lesion to the basal ganglia lesion is necessary to induce blepharospasm (Schicatano et al., 1997).

Thus, GMV studies have shown an involvement of primary and secondary cortical areas of frontal, parietal and temporal lobes in cervical dystonia (Draganski et al., 2003; Obermann et al., 2007) and in blepharospasm (Delmaire et al., 2007; Martino et al., 2011; Suzuki et al., 2011). Activation of the anterior cingulate cortex (ACC) is suggested with PET studies in hemidystonia and blepharospasm patients by increased GMV (Ceballos-Baumann et al., 1995a; Kerrison et al., 2003). A meta-analysis performed with the anatomic likelihood ratio estimation (ALE) method, comparing patients with primary focal dystonia and healthy controls, still showed an increased GMV in the caudate nucleus, the primary motor cortex (M1) and the post-central gyrus and a decreased GMV in the thalamus and the putamen in dystonic patients (Zheng et al., 2012). In patients with writer’s cramp, an increased glucose metabolic activity has been demonstrated in the childhood onset *DYT1* dystonia in the thalamus, midbrain and cerebellum using^18^F-fluorodeoxyglucose PET (Eidelberg et al., 1998), and in the lateral frontal cortex, the paracentral cortex bilaterally and the contralateral lentiform nucleus, pons and midbrain in idiopathic torsion dystonia (Eidelberg et al., 1995). Moreover, H_2_O^15^ PET studies have shown an over activity induced by joystick movement in freely selected directions paced by a tone at 3 Hz, in several ipsi- and contralateral cortical areas of five patients with acquired hemidystonia in comparison with control subjects, and in ipsilateral cerebellum (Ceballos-Baumann et al., 1995a). In line with these data, using magnetic resonance spectroscopy, a reduced level of GABA, which is the main inhibitory neurotransmitter in neurons, has been reported in the contralateral motor cortex and in the lenticular nucleus of seven writer’s cramp patients in comparison with healthy controls (Levy and Hallett, 2002) suggesting a loss of inhibition of these structures.

In those dystonic syndromes, conflicting data have been published considering the GMV of cerebellar lobes (Draganski et al., 2003; Delmaire et al., 2007; Obermann et al., 2007), but an increase of glucose metabolic activity of the cerebellum was evidenced in hereditary dystonia (Carbon and Eidelberg, 2009; Carbon et al., 2010) and an abnormal burst activity in Purkinje cells has been recorded in awake genetically dystonic rats (LeDoux and Brady, 2003). Moreover, high-field MRI diffusion tensor imaging images, used to reconstruct connectivity maps using probabilistic tractography, showed an impaired integrity of the cerebello-thalamo-cortical fiber tracts in early onset familial dystonia associated with *DYT1* or *DYT6* mutations (Argyelan et al., 2009). This observation seems to be particularly relevant, since this impaired circuitry was also present, although to a lesser extent, in asymptomatic dystonia mutation carriers in whom it was correlated with an increased motor activation evaluated by O^15^PET imaging, consistent with a loss of cerebellar inhibition on the motor output.

Sensory cortex and sensorimotor integration have gained much importance in the pathophysiological models of dystonia. Indeed, sensory tricks (sensory stimulus) able to neutralize dystonic postures are commonly reported in primary dystonia and suggest an alteration of the sensory input processing. Indeed, sensory training with Braille reading improves spatial acuity but also the Fahn dystonia scale in focal hand dystonia patients (Zeuner et al., 2002). Even if patients with dystonia usually do not have sensory symptoms or clinical abnormalities at neurological examination, mild sensory dysfunction such as the increase of the somesthesic temporal discrimination threshold has been described (Tinazzi et al., 1999; Naumann et al., 2000). Brain imaging studies have shown an increased GMV of the hand representation area of primary somatosensory cortex (SSC) in focal hand dystonia (Garraux et al., 2004), and increased cerebral blood flow using H_2_^15^O-PET (Ceballos-Baumann et al., 1995a,b). The impact of tactile discrimination in writer’s cramp patients (Schneider et al., 2010) and blepharospasm (Schmidt et al., 2003) in comparison to controls was illustrated by a greater bilateral ventromedial pallidal activity. Also, dystonic movements can be induced in patients with writer’s cramp by a vibration stimulator (120 Hz; 1-2 mm amplitude) held in the palm during 2-3 min (Kaji et al., 1995). All these data suggest an important role of the sensory afferent pathway on the dysfunction of the cortico-striato-thalamo-cortical loop in that disease.

In summary, all these data have led to the “network pathophysiological model”, in which multiple lesions in and outside the basal ganglia and/or defective interactions among different nodes in the motor network can produce dystonia, with no overt cell loss (Jinnah and Hess, 2006), indicating he functional nature of the disease.

## Dystonia and TMS: A Window into Pathophysiological Insights

Transcranial magnetic stimulation (TMS) is a non-invasive technique of cortical neurons stimulation. The production of a short and intense current in a coil applied over the scalp generates a magnetic field perpendicular to the coil, which passes through the skull and induces an electric field parallel to the surface of the brain. Due to the induction of ions flux (Roth et al., 1991), depolarization of the most superficial, preferentially horizontally oriented interneurons occurs (Day et al., 1987; Di Lazzaro et al., 2000a). A single TMS pulse applied over the M1 generates descending volleys traveling in the pyramidal track that lead to a MEP in the target muscle. It evaluates the conduction through the corticospinal pathway and the cortical excitability by determining the MEP amplitude after various conditioning stimuli.

Multiple parameters have been used to measure cortex excitability in dystonia. The motor threshold (MT) is defined as the lowest TMS intensity sufficient to produce a MEP in the target muscle, in at least 50% of trials. MT is highly variable among individuals but remarkably constant in a given individual. The recruitment curve (RC), which represents the relation curve between the magnetic stimulus strength and the MEP amplitude obtained after stimulation of the M1 is used to assess the neurons membrane excitability. The cortical silent period (CSP) is obtained after an initial TMS suprathreshold stimulation over the M1 that induces a “pause” on the subsequent EMG muscle voluntary activity. This CSP originates mainly, at least for its late part, from the activation of cortical inhibitory interneurons presumably mediated by GABA-B receptors (Werhahn et al., 1999). Paired associative stimulation refers to a paradigm consisting of a single electrical stimulus directed to a peripheral nerve in advance of a single pulse TMS delivered to the contralateral M1. Repeated pairing, or association, of the stimuli may increase or decrease the excitability of corticospinal projections from M1, depending on the interval between the afferent stimulus and the magnetic pulse (Classen et al., 2004). Accordingly, if the ISI between the peripheral nerve stimulus and the TMS pulse is fixed at around 25 ms, it will generate a sustained increase in cortico-spinal excitability. Instead, if the ISI is fixed around 10 ms, it produces a reduction in cortico-spinal excitability (Carson and Kennedy, 2013). This inhibitory circuit is likely mediated by GABA-A (Di Lazzaro et al., 2007) and central cholinergic activity (Di Lazzaro et al., 2000b). Paired pulse stimulus is a protocol that assesses the excitability of intracortical circuitry using TMS over the M1. A “conditioning stimulus” is delivered prior to a “test stimulus”, at various ISI (Thickbroom et al., 2006), resulting in a decrease of the MEP amplitude, named intracortical inhibition (ICI), or increase of the MEP amplitude, named intracortical facilitation (ICF), compared to the TMS alone stimulation (Rossini et al., 2015). An initial subthreshold conditioning stimulus followed by a supramaximal test stimulation has an inhibitory effect on the motor cortex at short ISI <5 ms (SICI) considered of cortical origin (Kujirai et al., 1993), while intervals between 50 to 200 ms allow the recording of long ICI (Sanger et al., 2001). It has a facilitating effect (ICF) at longer ISI intervals, usually between 7 and 13 ms (Ziemann et al., 1996b; Di Lazzaro et al., 2000a). The paired pulse stimulation paradigm has been widely used to investigate the cerebello-thalamo-cortical pathway in which the cerebellar Purkinje cells inhibitory drive reduces the cerebellar dentate nucleus excitatory output to the motor cortex (Ugawa et al., 1995). Paired pulse stimulation in 8 healthy subjects showed that the MEP, induced by a contralateral motor cortex magnetic stimulation, were suppressed after a cerebellar conditioning test with an ISI of 5-8 ms, this inhibition being not found in two patients with cerebellar cortical atrophy and cerebellar hemispheres lesions, highly suggestive of the necessity of a functional cerebellar circuitry to inhibit the motor cortex in normal subjects (Ugawa et al., 1995).

### Surround Inhibition Is Suppressed

The balance between excitatory signals (to agonist’s muscles for example) and inhibitory signals (to antagonist’s muscles for example) that takes place during movement is considered as essential for the motor system to selectively direct the desired movement. It is suspected to be impaired in dystonia leading to muscles co-contraction. After a single TMS pulse in healthy volunteers, the amplitude of the MEP recorded on the adductor digiti minimi muscle of the little finger, which is innervated by the ulnar nerve, was suppressed during voluntary flexion of the index finger, which is innervated by the median nerve: this phenomenon is referred to the so-called surround inhibition (Sohn and Hallett, 2004b; Beck et al., 2008). Conversely, in seven patients with focal hand dystonia free of concomitant medication, the MEP recorded from the adductor digiti minimi, which was not involved in the desired task, was increased during flexion movement of the index finger in comparison with seven normal subjects (Sohn and Hallett, 2004a), which consequently suggests an altered surround inhibition. This seems particularly relevant in the pathophysiology of cervical dystonia, as surround inhibition is preserved in psychogenic dystonia (Quartarone et al., 2009), as well as in the asymptomatic arm uninvolved in the movement disorder (McDougall et al., 2015). The cortico-spinal conduction time is normal in non-primary dystonia secondary to a basal ganglia lesion (Terao et al., 1995), as well as in various forms of primary dystonia such as writer’s cramp (Mavroudakis et al., 1995; Rona et al., 1998), segmental dystonia (Rona et al., 1998), task specific dystonia (Ikoma et al., 1996), and fixed dystonia (Avanzino et al., 2008), in accordance with the absence of structural lesions of the corticospinal tract associated with the disease.

### Cortical Inhibition Is Lost

Loss of inhibition in dystonia has been shown through various electrophysiological paradigms, mainly through TMS parameters, but also using blink reflex and somatosensory evoked potentials (SSEP). The blink reflex, induced by an electrical stimulation of the supraorbital nerve, produces a reflex contraction of the orbicularis oculi. One short homolateral latency R1 through an oligosynaptic pathway in the pons and two long homolateral and contralateral latencies R2 are produced through a polysynaptic pathway via the lower brainstem (Kimura, 1970; Kimura and Lyon, 1972). Higher R1 and R2 amplitudes responses are interpreted as resulting from the hyperexcitability of the interneurons of the blink reflex pathway. In oromandibular dystonia, the reflex pathway is presumed to be structurally intact, as the latency of both R1 and R2 responses are normal. During voluntary contraction, the R1 and R2 amplitudes are significantly higher in comparison with control subjects, and have been interpreted as resulting from the hyperexcitability of the interneurons of the blink reflex pathway (Berardelli et al., 1985).

The SSEP, which are potentials detected by electroencephalography and evoked after a repeated somatosensory stimulus of the peripheral nerves, evaluate the integrity of the different structures along the somatosensory pathway. The amplitude of the N30 cortical component, presumably originating from the supplementary motor area (SMA) (Mauguière et al., 1983), informs on the activation of the SMA. In comparison with control subjects, the amplitude of the N30 was increased after median nerve stimulation in a group of 10 focal, segmental and generalized dystonias, whereas SSEP latencies were normal. This suggests an abnormal activation of the SMA on both sides without a structural lesion of the lemniscal pathway (Reilly et al., 1992). These results are concordant with the activation of the SMA found on FDG-PET studies (Ceballos-Baumann et al., 1995a). Moreover, the amplitudes of the P22/N30 precentral SSEP component, recorded at the frontal or central scalp electrodes, were significantly higher on the opposite side of the torticollis of 40 patients with cervical dystonia compared to healthy controls (Kanovský et al., 1997), showing in this case a lateralization of the cortical activation.

Transcranial magnetic stimulation only has allowed giving a deeper insight into the loss of cortical inhibition. The CSP evaluation in dystonia has not produced consistent results. Most studies report a decrease (Chen et al., 1997; Filipović et al., 1997; Rona et al., 1998; Pirio Richardson, 2015), or a trend toward a reduction of CSP (Schwenkreis et al., 1999), interpreted as a correlate to the cortical hyperexcitability, more rarely unchanged (Stinear and Byblow, 2004). Furthermore, to assess the neurons membrane excitability in dystonia, the MT, and the RC (Ziemann et al., 1996a; Chen et al., 1997; Werhahn et al., 1999; Ziemann et al., 2004; Murase et al., 2005) were assessed and were consistently normal in both primary and non-primary dystonia (Quartarone et al., 2009; Kojovic et al., 2013). This last result suggests that dystonia is induced rather by a loss of inhibition of the motor circuitry rather than a modification of the neural membrane excitability.

Inhibitory intracortical mechanisms have been studied by measuring short-latency inhibition using paired-pulse TMS, in focal or segmental primary dystonia (Espay et al., 2006), focal hand dystonia (Ridding et al., 1995; Niehaus et al., 2001), cervical dystonia (Hanajima et al., 1998; Kanovský et al., 2003) and fixed dystonia (Avanzino et al., 2008). It showed abnormalities in the affected M1 or in both hemispheres. Furthermore, the low penetrance of *DYT1* hereditary dystonia allows the comparison of symptomatic and non-symptomatic carriers. In both cases, reduced cortical inhibition, as defined by reduced SICI and CSP, was demonstrated suggesting that the non-manifesting subjects have already subclinical physiological deficits (Edwards et al., 2003). These data are concordant with the finding of an increased cortical excitability in both abnormal and normal hemispheres in patients with unilateral dystonia (Ridding et al., 1995; Niehaus et al., 2001).

The role of the dorsal premotor cortex (PMC), upstream to M1, in the motor output, has been investigated in the pathology of focal dystonia using the paired-pulse TMS paradigm. A conditioning TMS was applied to the dorsal PMC and then a test pulse to the ipsilateral motor cortex, at an interval of 6 ms, in nine patients with cervical dystonia, compared to nine healthy subjects. It resulted in enhanced dorsal premotor–motor cortical inhibition, independent of the severity of the disease (Pirio Richardson, 2015). This enhanced inhibition is more consistent with an endophenotypic marker widespread in the brain, as it has been evidenced in the setting of task-specific dystonia, such as writer’s cramp (Pirio Richardson et al., 2014), and is hypothesized by the authors as compensatory to reduce abnormal motor output and sustained muscle contraction.

At last, an alteration of the modulation of the cerebellar cortex on motor cortex excitability in eight focal hand dystonia patients who underwent a conditioning cerebellar stimulus followed 5 ms after by the contralateral motor cortex stimulation or test stimulus, was evidenced by the absence of effect on MEP amplitude, SICI or ICF in contrast to the eight normal controls (Brighina et al., 2009).

### Cerebral Plasticity Is “Over-Adapted”

Plasticity, defined as the modifications occurring in a neuronal network induced by repeated stimuli, has been suspected to be altered in dystonia based on the observation that task specific dystonia occurred in patients during highly skilled repetitive movements. The experimental model was obtained in two monkeys highly trained for a specific hand movement until their performance accuracy dropped below 50% presumably mimicking a task specific dystonia. An electrophysiological mapping study of the representation of the hand revealed a degraded primary somatosensory cortical area (Byl et al., 1996). These findings were substantiated by paired associative stimulation experiments in human dystonia. An increase of MEP amplitude in the target hand muscles of writer’s cramp patients compared to controls was shown, demonstrating the exaggerated responsiveness to the conditioning stimulus (Quartarone et al., 2003). Similar findings were observed in the primary SSC with a temporarily facilitation of the cortical SSEP P27 component in a group of focal hand dystonia (Tamura et al., 2009), suggesting an involvement of the primary SSC in dystonia. Furthermore, blocking muscle sensory afferents by intramuscular injection of lidocaine or ethanol in clinically involved muscles improves symptoms in writer’s cramp patients (Kaji et al., 1995). In healthy non-musician subjects, vibration increased the amplitude of motor-evoked potentials and decreased the short-latency (SICI) in the vibrated first dorsal interosseous muscle, whilst having the opposite effect on the non-vibrated adductor digiti minimi, as expected considering the surround inhibition effect. By contrast, in musician’s dystonia, vibration reduced SICI in all hand muscles, suggesting a disruption of the spatial organization of the interactions between sensory input and motor output to intrinsic hand muscles is this population. The authors hypothesized that in susceptible individuals, an initially beneficial adaptation of sensorimotor organization may “over develop” and lead to problems in targeting motor command (Rosenkranz et al., 2005). Moreover, using the Paired Associative Stimulation protocol in which a TMS pulse is delivered to the contralateral M1 25 ms after a conditioning stimulus directed to the median nerve, 10 patients with various forms of organic dystonia (writer’s cramp, cervical dystonia, blepharospasm, or oromandibular dystonia) showed an enhanced facilitation of MEP both in the median innervated abductor pollicis brevis, but also in the ulnar innervated first dorsal interosseus, while healthy subjects and psychogenic dystonia had normal facilitation in the abductor pollicis brevis and absent facilitation in the first dorsal interosseus. Alltogether, these data suggest that organic dystonia patients have a tendency to strengthen sensori-motor associations, resulting in the formation of unwanted muscle contractions and clinical dystonia (Quartarone et al., 2009).

In summary, dystonia may be characterized by two main pathophysiological abnormalities: “reduced” excitability of inhibitory systems at many levels of the sensorimotor system, and “increased” plasticity of neural connections in sensorimotor circuits (Quartarone et al., 2009) resulting in an overall cortical hyperexcitability. The relevance of such excitability abnormalities and underlying compensatory networks in the physiopathology of dystonia is critical. Indeed, we will see in the next chapter how these can be exploited by therapeutically strategies. Also, very interestingly, these excitability modifications might be a biomarker of patients who could be more sensitive to the excitatory modulating therapeutics.

However, most of these studies are hampered by the small sample size, the heterogeneity of dystonic patients evaluated in the same study or between similar studies, the absence of proper sham conditions (see below) and/or the absence of strict control of coil stimulation position, which precluded direct comparison between them or the generalization of the findings.

## Therapeutic Procedures In Dystonia

Repetitive application of magnetic pulses (repetitive TMS: rTMS) can be used to positively or negatively modulate cortical excitability beyond the stimulation period. Conventional rTMS consists of trains of usually biphasic magnetic pulses delivered at various frequencies. High-frequency stimulation (>1 Hz) trains, are administered discontinuously (Rossi et al., 2009). They are associated with an increased excitability demonstrated by increased MEP amplitude when applied over the M1. They have been correlated with an increased blood flow and glucose metabolism of the stimulated cortical area, when performed over the primary motor or mid-dorsolateral frontal cortex (Siebner et al., 2000; Paus et al., 2001). Low-frequency stimulation consists of a continuous train of pulses delivered at a frequency ≤1 Hz. It is associated with a transient decrease of cortical excitability demonstrated by decreased MEP amplitude when applied over the M1 (Chen et al., 1997), or over the dorsal PMC, which has been correlated to a widespread bilateral decrease of the cerebral blood flow, and by “extension”, of neuronal activity of prefrontal, premotor, M1, and left putamen, which was increased in the cerebellum. The after effect lasts one hour after the end of stimulation (Siebner et al., 2003).

Theta burst stimulation corresponds to modified rTMS protocols. They are characterized by repetitive sequences of a few high frequency pulses (∼3 pulses at 50 Hz) repeated at frequencies in the range of the EEG theta rhythm (4-7 Hz). They can be continuous (cTBS), or intermittent (iTBS). Following cTBS, MEP are suppressed, whereas following iTBS they are facilitated. Their effect can last up to one hour after their delivery (Huang et al., 2005).

Transcranial direct current stimulation delivers weak polarizing direct currents to the cortex. It is polarity dependent: anodal stimulation increases the network excitability and cathodal stimulation decreases the network excitability (Nitsche and Paulus, 2001).

A greater and longer after-effect that can last two weeks after the repeated rTMS sessions compared to the shorter after-effect after a single rTMS session, has been shown in various neurological diseases, such as chronic pain suggesting the increased benefit of repeated rTMS sessions (Khedr et al., 2005). The mechanisms underlying the after-effect modulation of rTMS are not completely understood. LTP has been initially evidenced in the rabbit hippocampus by an increase of the potential amplitudes recorded in post synaptic neurons following a high-frequency train of electric stimulation of the afferent pathway (Bliss and Lomo, 1973). On the contrary, long-term depression (LTD) can be induced at low frequency stimulation. The molecular basis of the synaptic plasticity of both LTP and LTD involves N-methyl-D-aspartic acid (NMDA) and *y*-aminobutyric acid (GABA) signaling (Collingridge et al., 1983; Dudek and Bear, 1992; Mulkey and Malenka, 1992; Tsien et al., 1996; Stagg et al., 2009). Furthermore, the blockage of LTP by intracellular injection of a calcium chelator suggests that LTP is related to post-synaptic neuron changes (Lynch et al., 1983; Mulkey and Malenka, 1992). Moreover, rTMS has been associated with neurotrophic effects. Daily rTMS sessions (20 Hz, 150 stimuli) up-regulate brain derived nerve factor (BDNF) gene expression after one week (Müller et al., 2000) and promotes sprouting of mossy fibers of the hippocampus (Lisanby and Belmaker, 2000) in rats’ brain. The dendritic sprouting and increased synaptic contacts have been linked to the BDNF-tyrosine kinase B pathway (Ma et al., 2013). Moreover, high frequency rTMS has been associated with enhanced hippocampal neurogenesis in rats (Ueyama et al., 2011). In humans, rTMS over the temporal auditory cortex increases GMV within the first week of stimulation in the temporal area contralateral to the site of stimulation. Due to the temporal relationship between changes of the patients’ auditory evoked potentials and these GMV changes, they are thought to be related to modifications in the synaptic strength (May et al., 2007).

Since rTMS induced currents are restricted to the two most superficial centimeters of the brain (Bohning et al., 1997), it precludes the direct stimulation of the basal ganglia. However, as a safe and non-invasive technique, rTMS efficacy was tested on dystonia especially based on the assumption that there is an increased cortical activity that could be potentially inhibited by low inhibitory rTMS protocols. However, studies evaluating the therapeutic benefit of rTMS in dystonia are still scarce. According to the pathophysiology mechanisms developed above, rTMS procedures have been successfully applied over the primary and PMC, the SSC, the ACC, and the cerebellar cortex.

### Primary Motor Cortex

Because dystonic patients present an increased excitability of the M1, the effects of one inhibitory 30-minute low-frequency 1Hz rTMS session applied over the left M1 were first assessed in writer’s cramp patients. In an unblinded randomized study, they induced a transient beneficial effect measured on handwriting. Six out of the seven patients had a reduced writing pressure twenty minutes after the end of the rTMS session that lasted at least three hours, and for two of them several days. This clinical improvement was associated with a normalization of the deficient cortico-cortical inhibition (Siebner et al., 1999). No modification was noticed after the sham stimulation with the active coil placed 2 cm anterior to Fz 10-20 electrode placement reference.

### PMC and SMA

A low-frequency inhibitory 1 Hz rTMS session applied over the PMC of healthy subjects in an open study was able to suppress the MEP recorded in a small hand muscle after a single pulse TMS stimulation of the motor cortex, indicating the reduction of M1 excitability located downstream the secondary motor area (Gerschlager et al., 2001). The effect of an inhibitory stimulation was assessed after a single session of 30 min low frequency 1 Hz rTMS applied over the contralateral PMC in eight writer’s cramp patients, in order to reinforce the inhibitory output of the PMC as seen above. Improved handwriting velocity was observed at 10 and 40 min while reduced handwriting discomfort was only noticed at 10 min (Tyvaert et al., 2006). The benefit of an inhibitory rTMS session was thus compared in three different cortical areas in focal hand dystonia including the PMC. In this single-blind study, the effect of a single 20 min 0.2 Hz rTMS session (250 monophasic pulses) was evaluated after application over either M1 or PMC or the SMA and was compared to a sham stimulation in 9 patients. A beneficial effect on computer aided rating of handwriting was demonstrated when stimulation was applied over the PMC but not when it was applied over M1, the SMA or after sham stimulation. This rTMS protocol was associated with an increased cortical inhibition measured by a prolonged CSP after the session. This study suggests a superiority of rTMS application over the PMC. However, the absence of stereotaxic localisation precludes the possibility to precisely know where the rTMS was applied. Therefore, the observed improvement could be due to the presumed inhibitory effect of rTMS over the PMC or over the nearby M1 (Murase et al., 2005). However, in an open study, an inhibitory cTBS protocol (40 s of uninterrupted application of a series of three stimuli delivered at 50 Hz repeated at intervals of 200 ms) applied on left PMC in eight writer’s cramp did not allow a recovery of the loss of surround inhibition evaluated by cortical excitability of the first dorsal interosseus, compared to baseline condition, while the writing performance was improved (Veugen et al., 2013). These results suggest that surround inhibition recovery is not the key factor for clinical improvement, or that the involvement of the PMC in the genesis of surround inhibition was limited in these patients. However, a single cTBS session might not have been sufficient to restore surround inhibition, in contrast to the efficient effect of cumulative daily sessions of cTBS applied over the PMC of focal hand dystonia patients (Huang et al., 2010).

A double-blind randomized, sham controlled study (double 70 mm air cooled coil identical to the active coil producing a click), evaluated the effect of rTMS modulation over four left cortical areas (motor cortex, dorsal PMC, SMA, ACC) in eight patients with cervical dystonia. Each subject underwent five 15-min low frequency rTMS session (180 pulses). Each rTMS session was processed at least two days apart, the order of which being randomly assigned. Potentially confounding concomitant medications were excluded with the exception of benzodiazepines. The rTMS stimulation was stereotaxically guided allowing the precise determination of the stimulation site localisation by the use of extra or intracranial landmarks. The clinical evaluation, based on the Toronto Western Spasmodic Torticollis rating Scale (TWSTRS), was performed before and after each session. Lower TWSTRS scores, indicating improvement, were observed after rTMS over the premotor and motor cortical areas (Pirio Richardson, 2015). These results in cervical dystonia are in line with the efficacy of targeting the premotor and the motor cortices in focal hand dystonia (Siebner et al., 2003; Murase et al., 2005; Borich et al., 2009; Kimberley et al., 2013). No definite conclusion can be drawn from the lower efficacy of rTMS session over the ACC, which was possibly improperly targeted by the figure-8 coil. No adverse event was reported.

Multiple rTMS sessions were then performed to assess a potential long lasting effect. Inhibitory low-frequency 1Hz rTMS was applied in six patients with focal hand dystonia over the contralateral PMC for five consecutive days (900 monophasic pulses). In a “single-blind” partially cross-over study (subjects in the initial active treatment group did not receive the sham session), the results of a digitalized handwriting test were compared between active rTMS and sham stimulation (90° angled coil). Prior to the rTMS sessions, focal hand dystonia patients had a reduced CSP. Across the five days of rTMS session, the pen velocity of rTMS treated patients improved in comparison to patients with sham stimulation and was maintained during the 10 days of follow-up. Furthermore, 2/3 of the patients reported a subjective improvement lasting 10 days. This improvement was associated with a prolongation of CSP (Borich et al., 2009). Similarly, a pilot study was conducted in a single patient with primary cervical dystonia and writer’s cramp, submitted to an inhibitory low-frequency 1 Hz rTMS sessions of 20 min during 5 days over the left PMC. A clinical evaluation was performed based on the Burke, Fahn, and Mardsen (BFM) rating scale and a global improvement scale. The BFM score did not improve either immediately or after 1 and 4 months after the last rTMS session. However, the cervical subset of the BFM score improved by 50% and was sustained for 4 months (Allam et al., 2007).

The effect of rTMS in non-primary dystonia was investigated in a single open study. In a series of three patients with unilateral or bilateral lesions of basal ganglia and in whom dystonia treatments were not discontinued, a daily session of a 20 min 1 Hz rTMS (1200 pulses) applied over the left PMC during five days was clinically evaluated. The average number of painless spasms was reduced in the three patients while the intensity pain scale was reduced in two patients 6 h after the last rTMS session in comparison to baseline (Lefaucheur et al., 2004). These results need to be confirmed by a randomized study investigating a larger population.

Also, such excitability abnormalities in the physiopathology of dystonia might also be prognostic markers of response to rTMS. Indeed, in a pilot study of a 6 week session of inhibitory low frequency rTMS administered over the contralateral dorsal PMC of two focal hand dystonia patients, only the patient with reduced CSP and SICI, which reflects a loss of cortical inhibition, had clinical benefit from rTMS while the other patient without CSP and SICI reduction did not improve (Kimberley et al., 2015a).

### Somatosensory Cortex

The SCC was the next second potential target for the treatment of dystonia, based on the improvement of tactile discrimination after an excitatory 5 Hz rTMS protocol applied over the primary SSC in healthy subjects in an open study (Ragert et al., 2003) and based upon alteration of the sensorimotor integration in dystonia, the increased GMV of the SSC in focal hand dystonia (Garraux et al., 2004) and the alteration of the digit representation on fMRI in the SSC in focal hand dystonia patients (Butterworth et al., 2003). An excitatory high frequency 5 Hz-rTMS was applied over the contralateral primary SSC administered by trains of 50 pulses (1250 pulses) in five writer’s cramp patients. Although it was associated with an increased hemodynamic response of the stimulated S1 cortex, it failed to improve patients’ tactile discrimination in comparison to sham stimulation, in dystonic patients, contrarily to controls subjects (Schneider et al., 2010). In controls, concomitant fMRI showed increased activity of the stimulated S1, bilateral PMC and basal ganglia, whereas, fMRI showed similar cortical effects to controls except for no effects in basal ganglia of dystonia patients. These data suggest a possible defective connection between both sites in the dystonic patients of that study (Schneider et al., 2010).

In order to modulate this aberrant functioning of the SSC, a cross-over single-blind four-week study of a 30 min 1 Hz rTMS session applied over the SSC during five consecutive days each week in 15 right handed writer’s cramp patients was evaluated versus sham stimulation (coil angled at 90°) at the end of each week (day 5, 14, and 28), the coil being maintained with a gripping arm. The SSC was localized with fMRI and the coil position was determined as the higher activation spot on the contralateral primary SSC of the hand (S1) after a passive simple movement of the hand. In the four patients with a subjective and objective clinical therapeutic benefit, the rTMS proved to have been administered on a narrow strip over the post central sulcus of the S1 sulcus. In the 11 non-responders patients, the rTMS had been administered outside this cluster suggesting that a precise localization of the coil is critical. Furthermore, in the four clinically improved patients, fMRI analysis underwent the week after rTMS, showed that simple passive movement used for the determination of the adequate rTMS coil position was associated with an activation of the posterior parietal cortex, the SMA on both sides and of the right anterior insula. This improvement was maintained during the three following weeks (Havrankova et al., 2010). The normalization of the sensorimotor integration, as demonstrated by the increase to normal values of the short afferent inhibition as measured with a sensory conditioning stimulus applied with ring electrodes at the index finger (Zittel et al., 2015), after an inhibitory 1Hz rTMS session targeting the SSC of nine cervical dystonia patients, in a randomized controlled trial, supports the therapeutic effect of inhibitory 1 Hz rTMS of the SSC in dystonia patients.

### Anterior Cingulate Cortex

The ACC has been shown to project bilaterally to the facial nucleus in animal models (Morecraft et al., 2001). Furthermore, activation of the ACC has been demonstrated with PET studies in hemidystonia and blepharospasm patients in whom ACC GMV was increased (Ceballos-Baumann et al., 1995a; Kerrison et al., 2003). In seven patients with blepharospasm, a preliminary study evaluated the benefit of various cortical stimulation areas (ACC, PMC and SMA) on eye blink rate, the number of sustained blinks and time to eye closure. An inhibitory low-frequency rTMS (0.2 Hz) stimulation over ACC during 15 minutes (180 stimuli), proved, in a randomized controlled study, to produce a higher clinical benefit than M1 stimulation (Kranz et al., 2009). A prospective randomized versus a sham coil stimulation (silent sham-coiled + active coiled angled 90°) study, using a H shaped coil, specially designed to reach deep brains structures, stimulating with the inhibitory rTMS protocol described above, showed a trend toward a transient clinical benefit in blepharospasm patients. It was associated with a trend toward an improvement of the blink reflex recovery, defined by the reduction of the amplitude ratio of a test R2 response of the blink reflex over the conditioning R2 response administered 0.2 ms earlier. As basal ganglia have an inhibitory effect on the excitability of the trigeminal blink reflex (Basso and Evinger, 1996), these results indicate that effective rTMS applied to the ACC is associated with a reduction of the facial motoneurons and/or bulbar interneurons excitability possibly by recovery of the inhibitory control of the basal ganglia (Kranz et al., 2010).

### Cerebellar Cortex

Because of the loss of inhibitory control of the cerebellum over the motor cortex in dystonic patients (Siebner et al., 1999), emerging rTMS strategies target the cerebellum. Conflicting results have been obtained using tDCS. Anodal tDCS over the cerebellum showed a beneficial effect in eight focal hand dystonia patients (Bradnam et al., 2015). On the contrary, a single session of anodal tDCS applied over the cerebellum in 10 patients with writer’s cramp, failed to improve writing ability (Sadnicka et al., 2014). Due to the reduced modulation of the cerebellar cortex in motor cortex excitability in focal hand dystonia (Brighina et al., 2009), the effect of a TBS protocol over the cerebellar cortex was evaluated in idiopathic cervical dystonia in which reduced Purkinje cells has been documented (Prudente et al., 2013) and GMV increased (Draganski et al., 2003). An excitatory TBS protocol consisting of blocks of three pulses delivered at 50 Hz and administered every 200 ms (5 Hz) during five consecutive days for a 2-week period was delivered bilaterally over the cerebellar cortex in 20 right-handed patients with cervical dystonia. Patients were evaluated before the start of the iTBS protocol, the week following the two-week active treatment session or the sham stimulating session (coil angled at 90°) and two and four weeks after the stimulation period. A clinical improvement was demonstrated on a dystonia clinical rating scale 1 week after rTMS, which disappeared thereafter. Improvement was associated with the disappearance of the first dorsal interosseus potentiation evaluated with a Paired Associative Stimulation protocol. On the contrary, rTMS had no effect on MT and on SICI and ICF parameters in a randomized sham controlled study, suggesting that cerebello-cortical inhibition and motor cortex excitability are dissociated (Koch et al., 2014).

### Comparison of rTMS Protocols

The evaluation of motor function in combined therapy associating rTMS and rehabilitation was studied in nine patients with focal hand dystonia who were randomly assigned to a 20-min 1 Hz rTMS session (1200 pulses) applied during five consecutive days on the contralateral PMC in association either with 5 days of retraining (supervised practice of sensory discrimination) or 5 days of control therapy (active and passive generalized stretching to wrist) in a cross-over study with a washout period of 1 month. The global rating score improved in the two study groups throughout the study with an efficacy maintained until the second follow-up period (1.5-month post inclusion) showing the benefit of repeated rTMS sessions on the duration of rTMS beneficial effect. However, this study did not demonstrate an additional benefit of sensorimotor retraining, some patients being improved while other patients remained stable or deteriorated, suggesting that the appropriate timing of both therapies remains to be determined (Kimberley et al., 2015b).

A single pilot study used different rTMS protocols in the same group of patients. An inhibitory low-frequency rTMS (0.2 Hz) protocol during 15 min (180 stimuli) applied on the ACC, induced a clinical and electrophysiological (blink reflex recovery) benefit in a group of 12 blepharospasm patients while a 40 min iTBS protocol consisting of three TMS pulses at 50Hz delivered every 200 ms did not (Kranz et al., 2010). The presumed advantage of TBS protocols was therefore not demonstrated. However, the statistical analysis in this study was based on the mixed results of two times points and four different stimulation areas precluding definite conclusion on the observed results.

### Sham Stimulation

An important limitation in the interpretation of rTMS studies is the lack of optimal control condition. Sham intervention refers to the techniques used as a control in studies where a medical or surgical procedure constitutes the treatment. Besides its cortical effects, active rTMS stimulation is associated with perceptible effects such as the production of a clicking sound secondarily to the current flow in the coil and with trigeminal afferences stimulation underneath the coil resulting in somatosensory effects. Various sham procedures have been developed, none of which are perfect. Coils applied to the scalp with an angle have initially been used. In animal studies, substantial decrease in integrated voltage measured by stereotaxically implanted intracranial electrodes was found with 90° coil tilting but not with 45° coil tilting indicating that the 45° coil tilting procedure could thus not be considered biologically inactive (Lisanby et al., 2001). Similarly, magnetic stimulations delivered with the coil in various tilted positions demonstrated that none of the sham positions tested were ideal sham due to the possibility of slight cortical activation as measured by the integrated voltage recorded with intracranial electrodes (Lisanby et al., 2001) and due to a lesser scalp sensation (Loo et al., 2000). Furthermore, the 90° coil tilting and the shield equipped coils that reduce the effective magnetic field by approximately 80% fail to evoke somatosensory sensation perceived with the active TMS (Duecker et al., 2013). To reproduce click sound and somatosensory sensations, new sham coils have been manufactured, looking alike active coils but equipped with a magnetic shield resulting in a delivered magnetic field of only 10% compared to active coils (Sommer et al., 2006). These new coils seem to be more appropriate for sham rTMS procedures (Rossi et al., 2007) but this point needs to be confirmed.

### Safety Issues

Repetitive TMS is a safe technique as long as recommendations are followed. The frequency is similar in adults and in children and adolescent in whom their incidence is 1.2% (Krishnan et al., 2015). None of the rare adverse events associated with rTMS have been reported so far in dystonia patients submitted to rTMS. Seizures are reported in 0.62% of patients. Safety in non-epileptic patients depends on several stimulation parameters including stimulation intensity, duration of stimulation, frequency of stimulation, inter trains intervals and interval between rTMS sessions for the most common ones (Rossi et al., 2009). In epileptic patients, the prevalence rate of epileptic seizures is reported to be between 0 to 3.6%. Several clinical and electroencephalographic precipitating factors have been identified, such as complex temporal seizures, rTMS delivered after a recent seizure, interictal epileptiform discharges on surface EEG that all seem to favor rTMS related seizures. Syncopes have also been rarely reported (0.62%). Other sides effects, most often transient, include headache (11.5%), scalp discomfort (2.5%), and a variety of other even rarer effects (mood changes, twitching, itching, fatigue, dizziness, neck stiffness, neck pain, tinnitus sleepiness, nausea…). Proper patients’ selection is critical to avoid side effects. Especially concomitant potentially epileptogenic medications and parameters of stimulation are major concerns (Rossi et al., 2009).

## Perspectives

The use of TMS to gain insight into the pathophysiology of dystonia has paved the way for the development of rTMS protocols used a therapeutical tool. Studies of rTMS have mainly focused on primary dystonia due to their highest prevalence and the absence of overt structural brain lesion. The place of rTMS in secondary dystonia has yet to be evaluated. TMS has allowed better delineation of the mechanisms involved in the pathophysiology of dystonia. Loss of cortical inhibition, presence of sensorimotor integration and synaptic plasticity has been associated with dystonia. The lack of consistency observed between studies can be attributed to small sample sizes, different patient phenotypes sometimes included in the same study, heterogeneous study designs and differences in patient age and gender. Few studies have used strereotaxically guided techniques to position the coil adequately and limited data regarding the efforts made to maintain an adequate coil position during the entire rTMS session are available. These are probably factors that strongly limit the interpretation of rTMS results. Furthermore, data regarding confounding factors of cortex excitability, especially preliminary exercise, time of the day and concomitant medications (Ridding and Ziemann, 2010), are rarely mentioned. Nevertheless, preliminary results on the therapeutic efficacy of rTMS in the treatment of dystonia are promising. Further studies are needed to determine the most adequate site for rTMS administration, and to define the best protocol. Multicentre approaches are probably necessary to improve the statistical power of the results. Considering the long lasting effects of botulinum toxin and the time consuming nature of rTMS, which seems difficult to extend to routine practice, a serious concern is how to significantly prolong its positive after-effects, more than the usual 2 weeks obtained with TBS protocols or repetitive rTMS sessions (Siebner et al., 1999). At present, however, rTMS could be used to evaluate future responders to invasive surgical stimulator implantation.

## Author Contributions

All authors listed, have made substantial, direct and intellectual contribution to the work, and approved it for publication.

## Conflict of Interest Statement

The authors declare that the research was conducted in the absence of any commercial or financial relationships that could be construed as a potential conflict of interest.

## Abbreviations

ACC: anterior cingulate cortex
CSP: cortical silent period
fMRI: functional MRI
GMV: gray matter volume
ICF: intracortical facilitation
ISI: inter-stimulus interval
LTD: long term depression
LTP: long term potentiation
M1: primary motor cortex
MEP: motor evoked potential
MRI: magnetic resonance imaging
PET: positon emission tomography
PMC: premotor cortex
rTMS: repetitive transcranial magnetic stimulation
SICI: short intracortical inhibition
SMA: supplementary motor area
SSC: somatosensory cortex
SSEP: somatosensory evoked potentials
TBS: theta burst stimulation
tDCS: transcranial direct current stimulation
